# Persistent CRP Elevation at 4 Weeks Is Associated with Delayed Union After Polytrauma: An Exploratory Retrospective Cohort Study

**DOI:** 10.3390/diagnostics16121845

**Published:** 2026-06-15

**Authors:** Eduard Catalin Georgescu, Ioana Anca Badarau, Alexandru Lisias Dimitriu, Elisa Georgiana Popescu, Monica Georgiana Roman, Liliana Mirea, Dragos Ene, Razvan Ene

**Affiliations:** 1 Department 14—Orthopaedics and Intensive Care, “Carol Davila” University of Medicine and Pharmacy, 050474 Bucharest, Romania; eduard-catalin.georgescu@drd.umfcd.ro (E.C.G.); elisa-georgiana.popescu@umfcd.ro (E.G.P.); monica-georgiana.roman@drd.umfcd.ro (M.G.R.); liliana.mirea@umfcd.ro (L.M.); razvan.ene@umfcd.ro (R.E.); 2 Department of Orthopaedics, Clinical Emergency Hospital Bucharest, 050474 Bucharest, Romania; 3 Department of Physiology, “Carol Davila” University of Medicine and Pharmacy, 050474 Bucharest, Romania; anca.badarau@umfcd.ro; 4 Department of Anesthesiology and Intensive Care, Clinical Emergency Hospital Bucharest, 050474 Bucharest, Romania; 5 Department 10—General Surgery, “Carol Davila” University of Medicine and Pharmacy, 050474 Bucharest, Romania; dragos.ene@umfcd.ro; 6 Department of Surgery, Clinical Emergency Hospital Bucharest, 050474 Bucharest, Romania

**Keywords:** polytrauma, delayed union, fracture healing, C-reactive protein, interleukin-6, inflammatory biomarkers, long-bone fractures, mRUST

## Abstract

**Background/Objectives:** Delayed bone healing remains a relevant complication after polytrauma, where fracture repair occurs in the setting of systemic inflammation and repeated physiologic stress. This study evaluated whether serial changes in interleukin-6 (IL-6), C-reactive protein (CRP), and fibrinogen are associated with delayed union in polytrauma patients with long-bone fractures. **Methods:** We performed an exploratory retrospective cohort study including 115 adult polytrauma patients with long-bone fractures treated at a single tertiary trauma center between 2 January 2022 and 14 December 2024. Serum IL-6, CRP, and fibrinogen were recorded at 24 h, 72 h, 1 week, 2 weeks, and 4 weeks after injury. IL-6 was measured in the institutional clinical laboratory using routine immunoassay methods, whereas CRP and fibrinogen were measured using standard hospital analytical methods, including an immunoturbidimetric assay for CRP and the Clauss clotting method for fibrinogen. Radiographic healing was assessed at 6, 12, and 24 weeks using an mRUST-based healing score. The primary endpoint was clinician-assigned delayed union at 24 weeks; nonunion at 9 months was assessed secondarily. Complete-case multivariable logistic regression was performed in 86 patients, and exploratory longitudinal biomarker analyses used generalized estimating equations. **Results:** Delayed union at 24 weeks occurred in 39/115 patients (33.9%), while nonunion at 9 months occurred in 7/115 patients (6.1%). Patients with delayed union had longer time to definitive fixation (35.3 ± 10.2 h vs. 29.0 ± 14.0 h; *p* = 0.003) and more frequent shock on admission (43.6% vs. 23.7%; *p* = 0.047). IL-6 was higher in the delayed-union group at 1 week (57.3 ± 30.3 vs. 46.5 ± 29.2 pg/mL; *p* = 0.043) and 4 weeks (21.2 ± 11.6 vs. 17.1 ± 10.3 pg/mL; *p* = 0.022), whereas CRP was markedly higher at 4 weeks (29.4 ± 14.2 vs. 16.3 ± 10.6 mg/L; *p* < 0.001). After false-discovery-rate correction, only CRP at 4 weeks remained significant among serial biomarker comparisons. In multivariable analysis of 86 complete cases, CRP at 4 weeks remained independently associated with delayed union (adjusted OR 2.16 per 10 mg/L, 95% CI 1.36–3.43; *p* = 0.001). The model showed apparent discrimination with an AUC of 0.80 and acceptable calibration (Hosmer–Lemeshow *p* = 0.41). In sensitivity analysis excluding deep surgical-site infection cases, the association between CRP and delayed union persisted (adjusted OR 2.02 per 10 mg/L, 95% CI 1.26–3.26; *p* = 0.004). **Conclusions:** In this exploratory retrospective cohort of polytrauma patients with long-bone fractures, persistent post-traumatic CRP elevation at 4 weeks was associated with clinician-assigned delayed union, whereas IL-6 findings were weaker and exploratory. Because CRP is a nonspecific inflammatory marker, the observed association may reflect delayed healing, infection, reoperation, and/or persistent postoperative inflammatory burden. These data support association rather than validated prediction and require prospective validation with standardized outcome adjudication.

## 1. Introduction

Fracture healing after polytrauma is a complex biological process that unfolds in the setting of systemic inflammation, tissue hypoperfusion, blood loss, and repeated surgical stress. The host response to severe trauma includes an early pro-inflammatory phase that may be amplified by subsequent “second hits”, including operations, ischemia–reperfusion injury, and infection [1].

Immune signaling is integral to normal fracture repair, yet prolonged or dysregulated inflammation may impair the transition from early callus formation toward consolidation and remodeling [2]. IL-6 is a key cytokine within the early post-traumatic response, whereas CRP and fibrinogen represent clinically accessible acute-phase reactants that may reflect the persistence rather than merely the magnitude of inflammation. Experimental and translational work suggests that systemic immune signatures after trauma may help predict downstream bone regeneration [3].

Fracture repair after major trauma depends on a tightly regulated interaction between inflammatory signaling, angiogenesis, mechanical stability, and local tissue biology. Although an early inflammatory response is necessary for callus formation, prolonged systemic inflammation may impair the transition from soft callus to mineralized repair tissue and may contribute to delayed union or nonunion. This issue is particularly relevant in polytrauma patients, in whom fracture healing occurs in the context of hemorrhage, transfusion exposure, multiple injured organ systems, and staged operative treatment. In this setting, readily available circulating biomarkers may provide clinically useful adjunctive information beyond conventional radiographic follow-up.

Reliable outcome assessment is essential in studies of fracture healing. Radiographic union scoring systems, including RUST and mRUST, have shown good reproducibility and validity across several fracture-healing settings and are increasingly used to standardize follow-up evaluation [4,5,6,7]. Complementing radiographic surveillance with biological markers may improve early identification of patients at risk for impaired healing [8].

The objective of the present study was to explore whether serial post-injury changes in IL-6, CRP, and fibrinogen are associated with delayed union at 24 weeks in polytrauma patients with long-bone fractures. In contrast to studies focused primarily on early post-traumatic inflammation, we examined biomarker trajectories from 24 h to 4 weeks and related them to subsequent radiographic healing during follow-up. We hypothesized that persistent biomarker elevation beyond the immediate post-injury phase, particularly CRP in the subacute period, would be associated with delayed bone healing.

## 2. Materials and Methods

### 2.1. Study Design and Data Source

This retrospective observational cohort study was performed at the Department of Orthopaedics, Clinical Emergency Hospital Bucharest, Romania, using a prospectively maintained institutional workbook comprising three linked sheets: patient-level baseline variables, serial biomarker measurements, and radiographic follow-up assessments. The source dataset included adult polytrauma patients (age ≥ 18 years) with an index long-bone fracture and sufficient follow-up to determine healing status at 24 weeks. Patients were enrolled between 2 January 2022 and 14 December 2024, and follow-up data were available through 26 May 2025. Because the study relied on a retrospectively assembled clinical workbook, a prospective screening log of non-included patients was not available. For multivariable modeling, patients with missing IL-6 at 1 week and/or CRP at 4 weeks were excluded from the complete-case regression subset. This study consisted of a retrospective analysis of clinical, laboratory, and radiographic data collected as part of routine standard care in polytrauma patients. The research protocol for secondary use of these data was approved by the Ethics Committee of the Clinical Emergency Hospital Bucharest (approval no. 3570, approval date: 26 February 2025). The study is reported in accordance with the STROBE statement for observational studies.

#### Eligibility Criteria and Cohort Derivation

Patients were eligible for the primary cohort if they were adult polytrauma patients with an index long-bone fracture, baseline clinical data, serial biomarker sampling performed as part of routine care, and sufficient radiographic follow-up to classify healing status at 24 weeks. Because the source workbook represented an analytic clinical dataset rather than a prospective screening log, records not meeting these criteria were not available for reconstruction. For the complete-case multivariable model, exclusion was limited to missing IL-6 at 1 week and/or CRP at 4 weeks.

### 2.2. Operative Management

All patients underwent definitive operative fixation of the index fracture. Temporary external fixation was used in a subset of patients as part of staged management, after which definitive fixation was performed. Operative variables extracted from the source dataset included temporary external fixation (yes/no), definitive fixation type, and time from injury to definitive fixation in hours. For descriptive purposes, primary definitive fixation was defined as definitive fixation performed without temporary external fixation.

### 2.3. Variables and Follow-Up

Baseline variables included demographics, body mass index, smoking status, major comorbidities, injury severity indices, admission shock status, lactate, transfusion exposure, ICU admission, associated injuries, fracture characteristics, and fixation strategy. Fracture-level variables available in the source dataset included index fracture site, AO/OTA classification, open-fracture status, and Gustilo type for open fractures. However, more granular biomechanical variables such as fracture gap, reduction quality, fixation construct stability, and soft-tissue severity beyond open-fracture grading were not recorded in a standardized form. Serial serum IL-6, CRP, and fibrinogen were recorded at 24 h, 72 h, 1 week, 2 weeks, and 4 weeks after injury. IL-6 was measured in the institutional clinical laboratory using routine immunoassay methods, whereas CRP and fibrinogen were measured using standard hospital analytical methods, including an immunoturbidimetric assay for CRP and the Clauss clotting method for fibrinogen. Radiographic and clinical follow-up was available at 6, 12, and 24 weeks using an mRUST-based healing score, number of bridged cortices, pain at the fracture site, functional use/weight-bearing status, and radiographic classification. Postoperative complications were captured in the source dataset as binary outcomes (deep surgical-site infection within 90 days and reoperation by 6 months), but the exact dates of these events relative to biomarker sampling were not available.

### 2.4. Endpoints

The primary endpoint was delayed union at 24 weeks, defined according to the predefined healing-status category recorded at the 24-week follow-up in the source dataset. This classification was based on the radiographic healing assessment available at the 24-week visit and was interpreted alongside the mRUST-based healing score, the number of bridged cortices, pain at the fracture site, and functional use/weight-bearing status. Patients classified as having delayed union at 24 weeks were compared with those classified as having no delayed union. The source dataset did not record whether this classification required formal consensus among surgeons and/or radiologists, and a separate prespecified set of standalone clinical criteria for delayed union was not available beyond the follow-up variables captured in the dataset. Likewise, no single cortical-bridging threshold or mRUST cut-off was prespecified; rather, these elements informed the clinician-assigned healing-status category at 24 weeks. Blinding of radiographic assessors was not recorded in the source dataset. Accordingly, the primary endpoint should be interpreted as an exploratory clinician-assigned healing-status outcome rather than as a fully standardized radiographic endpoint. Secondary endpoints were the mRUST-based healing score over follow-up, clinical union at 24 weeks, and nonunion at 9 months.

### 2.5. Statistical Analysis

Continuous variables are presented as mean ± standard deviation in the main tables for comparability with prior orthopaedic and trauma literature, and medians with interquartile ranges were used in the complete-case comparison table. Normality was assessed using the Shapiro–Wilk test. Because several biomarker variables were not normally distributed, between-group comparisons used the Mann–Whitney U test for continuous variables and chi-square or Fisher’s exact tests for categorical variables, as appropriate. Univariable logistic regression was performed for selected candidate predictors of delayed union at 24 weeks. Variables entered into the multivariable model were selected a priori based on clinical relevance and data completeness and included age, shock on admission, open fracture, time to definitive fixation, IL-6 at 1 week, and CRP at 4 weeks. Biomarker effect sizes are reported per 10-unit increase for IL-6 and CRP and per 12 h increase for time to fixation. Complete-case multivariable regression included only patients with complete data for all model covariates; no imputation was performed. Multiple imputation was considered but was not performed because missingness affected the key biomarker covariates themselves, and the missing-at-random assumption could not be justified confidently in this retrospective, physiologically heterogeneous polytrauma cohort. Imputed sensitivity analyses were therefore not pursued. To explore potential selection bias, patients included in and excluded from the complete-case model were compared descriptively. Given 31 delayed-union events in the complete-case subset and six candidate predictors, the events-per-variable ratio was 5.2 and the multivariable analysis was considered exploratory. Model performance was summarized using the apparent area under the ROC curve (AUC), the Hosmer–Lemeshow goodness-of-fit test, and optimism-corrected AUC from 300 bootstrap resamples. Exploratory ROC analysis of CRP at 4 weeks alone was additionally performed to estimate a Youden-optimal threshold and the corresponding sensitivity and specificity. Collinearity diagnostics were assessed using variance inflation factors (VIFs) in the complete-case model. Given the exploratory design, limited event count, and low collinearity, we retained a parsimonious standard logistic model rather than introducing post hoc penalized regression. Because deep surgical-site infection and reoperation may confound interpretation of CRP and may represent downstream postoperative events rather than baseline predictors, prespecified sensitivity analyses excluded patients with deep SSI and, separately, patients with deep SSI and/or reoperation. Exploratory longitudinal biomarker trajectory analyses used generalized estimating equations with robust (sandwich) standard errors and an exchangeable working correlation structure on log-transformed IL-6, CRP, and fibrinogen values. Timepoint, delayed-union status, and their interaction were specified a priori to test whether biomarker trajectories differed by healing group. An exchangeable structure was selected a priori because the study comprised five protocol-defined follow-up timepoints over a relatively short interval and included incomplete repeated measurements; exploratory comparisons with an autoregressive structure yielded similar inferences and no meaningful improvement in QIC, so only the exchangeable-model results are reported. Exploratory serial biomarker *p*-values were additionally adjusted using the Benjamini–Hochberg false discovery rate procedure.

### 2.6. Use of Generative Artificial Intelligence

During manuscript preparation and revision, the authors used ChatGPT (OpenAI, GPT-4) for language editing and drafting support. No generative AI tool was used to generate or analyze the study data. All scientific content and final wording were reviewed and approved by the authors.

## 3. Results

### 3.1. Cohort Overview

Delayed union at 24 weeks occurred in 39 of 115 patients (33.9%), while nonunion at 9 months was observed in 7 patients (6.1%). The delayed-union and no-delayed-union groups were similar with respect to age, sex distribution, body mass index, and injury severity. However, patients with delayed union had a longer time to definitive fixation (35.3 ± 10.2 h vs. 29.0 ± 14.0 h; *p* = 0.003) and were more likely to present with shock on admission (43.6% vs. 23.7%; *p* = 0.047). Deep surgical-site infection within 90 days and reoperation by 6 months were also more frequent in the delayed-union group.

All 115 patients underwent definitive fixation of the index fracture. Temporary external fixation was used in 25 patients (21.7%), whereas 90 patients (78.3%) underwent primary definitive fixation without temporary external fixation. Definitive fixation consisted of intramedullary nailing in 71 patients (61.7%), plate fixation in 41 patients (35.7%), and circular frame fixation in 3 patients (2.6%). The overall time to definitive fixation was 31.2 ± 13.1 h (median 30.5 h). The index fracture site was the femoral shaft in 50 patients (43.5%), tibial shaft in 35 (30.4%), proximal tibia in 16 (13.9%), distal femur in 7 (6.1%), and tibial plateau in 7 (6.1%). Open fractures were present in 25 patients (21.7%) and were graded as Gustilo type I in 5 cases, type II in 7, type IIIA in 10, and type IIIB in 3. AO/OTA fracture classification was available descriptively, with 43 type A fractures (37.4%), 33 type B fractures (28.7%), and 39 type C fractures (33.9%), but was not incorporated into multivariable modeling because of sample-size constraints and concern for model instability. As shown in Table 1, patients with delayed union had a longer time to definitive fixation than those without delayed union.

For multivariable analysis, 86/115 patients (74.8%) had complete data for all model covariates. Missingness affected IL-6 at 1 week in 14/115 patients (12.2%) and CRP at 4 weeks in 18/115 patients (15.7%), with overlap in 3 patients; all other multivariable-model covariates were complete. Compared with included patients, those excluded from the complete-case model more frequently presented with shock on admission (48.3% vs. 24.4%; *p* = 0.029), whereas age, ISS, time to definitive fixation, delayed-union frequency, deep SSI, and reoperation rates were similar.

### 3.2. Serial Biomarker Profiles

**Figure 1:**
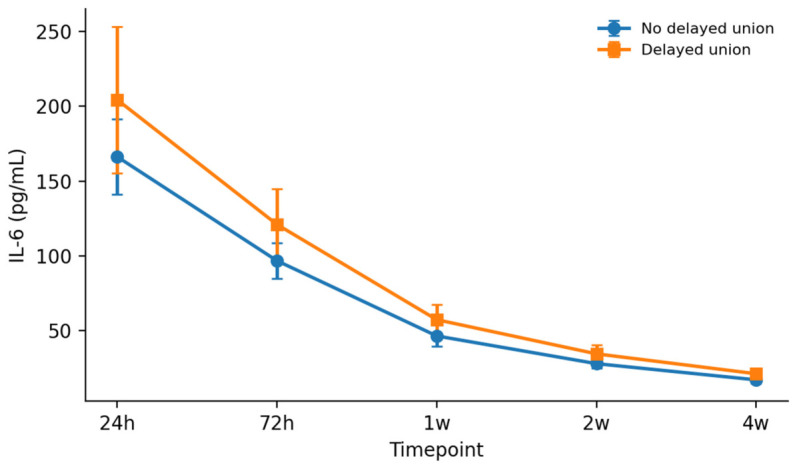
Serial IL-6 levels from 24 h to 4 weeks after injury according to delayed-union status. Error bars denote 95% confidence intervals. Exact *p*-values, *q*-values, and sample sizes at each timepoint are reported in Table 2.

**Figure 2:**
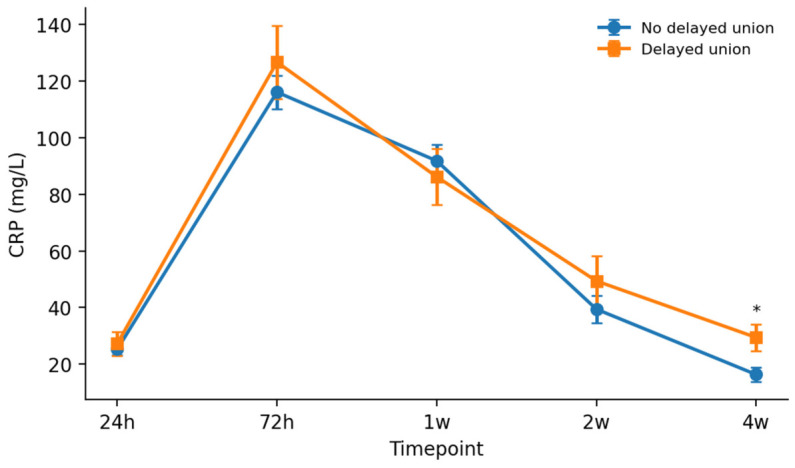
Serial CRP levels from 24 h to 4 weeks after injury according to delayed-union status. Error bars denote 95% confidence intervals. q < 0.05 after false-discovery-rate correction. Exact *p*-values, *q*-values, and sample sizes at each timepoint are reported in Table 2. * *p*-value < 0.001.

**Figure 3:**
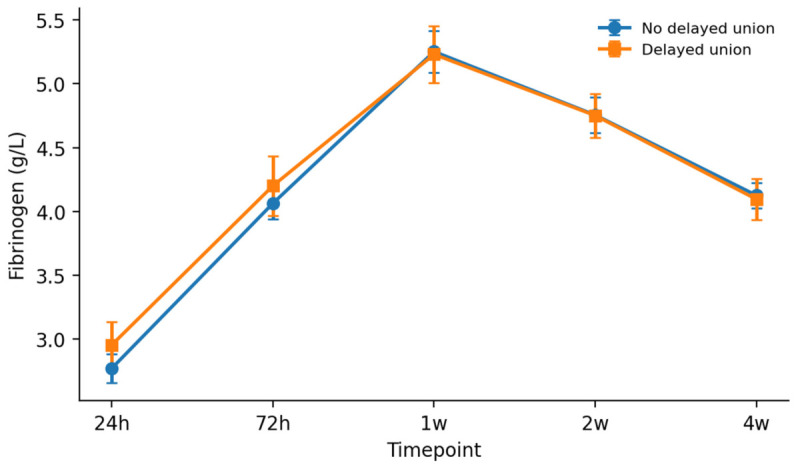
Serial fibrinogen levels from 24 h to 4 weeks after injury according to delayed-union status. Error bars denote 95% confidence intervals. Exact *p*-values, *q*-values, and sample sizes at each timepoint are reported in Table 2.

IL-6 values were consistently higher in the delayed-union group across all sampled timepoints and reached statistical significance at 1 week (57.3 ± 30.3 vs. 46.5 ± 29.2 pg/mL; *p* = 0.043) and 4 weeks (21.2 ± 11.6 vs. 17.1 ± 10.3 pg/mL; *p* = 0.022) in unadjusted comparisons. CRP showed the clearest between-group separation in the subacute phase, with a marked difference at 4 weeks (29.4 ± 14.2 vs. 16.3 ± 10.6 mg/L; *p* < 0.001). By contrast, fibrinogen followed the expected post-traumatic trajectory but did not differ significantly between groups at any assessed timepoint. After Benjamini–Hochberg false-discovery-rate correction across the 15 serial biomarker comparisons, only CRP at 4 weeks remained significant (q = 0.0001). In exploratory generalized estimating equation analyses using log-transformed biomarker values, the delayed-union group showed a significant CRP-by-time interaction at 4 weeks (β = 0.678, *p* < 0.001), whereas corresponding IL-6 and fibrinogen interaction terms were not significant. Exploratory autoregressive sensitivity models yielded the same qualitative conclusions. Serial biomarker trends with 95% confidence intervals are illustrated in Figure 1, Figure 2 and Figure 3.

**Table 2:** Serial inflammatory biomarker levels according to delayed-union status. Abbreviations: IL-6, interleukin-6; CRP, C-reactive protein. Values are presented as mean ± SD for comparability with prior orthopaedic and trauma literature; between-group comparisons used the Mann–Whitney U test. *q*-values were calculated using the Benjamini–Hochberg false discovery rate across the 15 serial biomarker comparisons.

Biomarker	Timepoint	No Delayed Union	Delayed Union	*p*-Value	*q*-Value
IL-6, pg/mL	24 h	166.3 ± 103.8 (*n* = 65)	204.4 ± 147.6 (*n* = 35)	0.298	0.423
IL-6, pg/mL	72 h	96.7 ± 49.3 (*n* = 68)	121.0 ± 68.5 (*n* = 32)	0.060	0.225
IL-6, pg/mL	1 w	46.5 ± 29.2 (*n* = 66)	57.3 ± 30.3 (*n* = 35)	0.043	0.215
IL-6, pg/mL	2 w	27.9 ± 12.2 (*n* = 67)	34.5 ± 17.2 (*n* = 34)	0.131	0.281
IL-6, pg/mL	4 w	17.1 ± 10.3 (*n* = 68)	21.2 ± 11.6 (*n* = 35)	0.022	0.163
CRP, mg/L	24 h	25.3 ± 8.7 (*n* = 68)	27.2 ± 12.8 (*n* = 35)	0.646	0.807
CRP, mg/L	72 h	116.1 ± 25.4 (*n* = 72)	126.7 ± 39.3 (*n* = 36)	0.275	0.423
CRP, mg/L	1 w	91.8 ± 23.4 (*n* = 63)	86.2 ± 27.9 (*n* = 31)	0.271	0.423
CRP, mg/L	2 w	39.4 ± 19.4 (*n* = 62)	49.4 ± 27.3 (*n* = 36)	0.107	0.281
CRP, mg/L	4 w	16.3 ± 10.6 (*n* = 62)	29.4 ± 14.2 (*n* = 35)	<0.001	<0.001
Fibrinogen, g/L	24 h	2.8 ± 0.5 (*n* = 67)	3.0 ± 0.6 (*n* = 36)	0.126	0.281
Fibrinogen, g/L	72 h	4.1 ± 0.5 (*n* = 73)	4.2 ± 0.7 (*n* = 35)	0.311	0.423
Fibrinogen, g/L	1 w	5.3 ± 0.7 (*n* = 71)	5.2 ± 0.7 (*n* = 33)	0.911	0.976
Fibrinogen, g/L	2 w	4.8 ± 0.6 (*n* = 66)	4.8 ± 0.5 (*n* = 36)	1.000	1.000
Fibrinogen, g/L	4 w	4.1 ± 0.4 (*n* = 66)	4.1 ± 0.5 (*n* = 35)	0.743	0.857

### 3.3. Radiographic Healing

**Figure 4:**
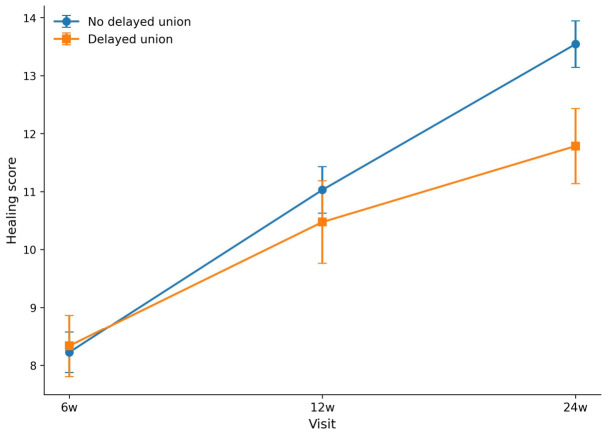
mRUST-based healing score at 6, 12, and 24 weeks according to delayed-union status. Error bars denote 95% confidence intervals. Exact *p*-values and sample sizes at each visit are reported in Table 3.

Radiographic progression was broadly similar between groups at 6 weeks. Follow-up completeness was 100% at 6 weeks, 94.8% at 12 weeks (109/115), and 94.8% at 24 weeks (109/115). Reasons for missing follow-up radiographs were not retained in the source dataset. Follow-up availability did not differ significantly between delayed-union groups at 12 weeks (71/76 vs. 38/39; *p* = 0.662) or 24 weeks (72/76 vs. 37/39; *p* = 1.000). By 12 weeks, patients with delayed union had fewer bridged cortices (2.2 ± 0.8 vs. 2.6 ± 0.7; *p* = 0.005). At 24 weeks, the delayed-union group had lower healing scores (11.8 ± 1.9 vs. 13.5 ± 1.7; *p* < 0.001) and higher pain at the fracture site (2.2 ± 1.3 vs. 1.6 ± 1.0; *p* = 0.018), consistent with less advanced fracture healing. The trajectory of radiographic healing scores is shown in Figure 4.

**Table 3:** Radiographic healing measures during follow-up according to delayed-union status. Abbreviations: mRUST, modified Radiographic Union Scale for Tibial Fractures; VAS, visual analogue scale.

Visit	Measure	No Delayed Union	Delayed Union	*p*-Value
6 w	Healing score	8.2 ± 1.5 (*n* = 76)	8.3 ± 1.6 (*n* = 39)	0.756
6 w	Bridged cortices (0–4)	1.5 ± 0.8 (*n* = 76)	1.7 ± 0.8 (*n* = 39)	0.243
6 w	Pain at fracture site (VAS)	5.2 ± 1.7 (*n* = 76)	4.7 ± 1.7 (*n* = 39)	0.079
12 w	Healing score	11.0 ± 1.7 (*n* = 71)	10.5 ± 2.2 (*n* = 38)	0.228
12 w	Bridged cortices (0–4)	2.6 ± 0.7 (*n* = 71)	2.2 ± 0.8 (*n* = 38)	0.005
12 w	Pain at fracture site (VAS)	2.7 ± 1.4 (*n* = 71)	3.2 ± 1.3 (*n* = 38)	0.079
24 w	Healing score	13.5 ± 1.7 (*n* = 72)	11.8 ± 1.9 (*n* = 37)	<0.001
24 w	Bridged cortices (0–4)	3.2 ± 0.6 (*n* = 72)	3.0 ± 0.9 (*n* = 37)	0.272
24 w	Pain at fracture site (VAS)	1.6 ± 1.0 (*n* = 72)	2.2 ± 1.3 (*n* = 37)	0.018

### 3.4. Regression Analysis

In univariable logistic regression, longer time to definitive fixation (OR 1.58 per 12 h, 95% CI 1.08–2.29; *p* = 0.017), shock on admission (OR 2.49, 95% CI 1.09–5.68; *p* = 0.030), and CRP at 4 weeks (OR 2.40 per 10 mg/L, 95% CI 1.58–3.64; *p* < 0.001) were associated with delayed union at 24 weeks. IL-6 at 1 week showed a weaker, non-significant association in univariable analysis. Complete-case multivariable analysis included 86 patients with 31 delayed-union events (events-per-variable ratio 5.2). In the multivariable model, CRP at 4 weeks remained independently associated with delayed union (adjusted OR 2.16 per 10 mg/L, 95% CI 1.36–3.43; *p* = 0.001), whereas time to definitive fixation, shock on admission, open fracture, age, and IL-6 at 1 week did not retain statistical significance. The model showed apparent discrimination with an AUC of 0.80, acceptable calibration (Hosmer–Lemeshow *p* = 0.41), and an optimism-corrected AUC of 0.75 after 300 bootstrap resamples. These values describe the apparent predictive performance of the exploratory model and should not be interpreted as evidence of formal validation. The apparent AUC of the multivariable model was 0.80 (95% CI 0.69–0.89). In exploratory ROC analysis of CRP at 4 weeks alone among patients with available 4-week CRP values, the AUC was 0.77 (95% CI 0.67–0.87). The Youden-optimal CRP threshold was 25.4 mg/L, corresponding to a sensitivity of 68.6% and a specificity of 80.6%; these values should be interpreted as descriptive and exploratory rather than clinically validated. No meaningful multicollinearity was detected (all predictor VIFs < 1.3). In a sensitivity analysis excluding patients with deep SSI from the complete-case subset, CRP at 4 weeks remained associated with delayed union (adjusted OR 2.02 per 10 mg/L, 95% CI 1.26–3.26; *p* = 0.004). Similar results were observed after excluding patients with deep SSI and/or reoperation (adjusted OR 1.92 per 10 mg/L, 95% CI 1.15–3.19; *p* = 0.012). Detailed regression and sensitivity results are presented in Table 4.

### 3.5. Complete-Case Comparison and Sensitivity Analyses

**Figure 5:**
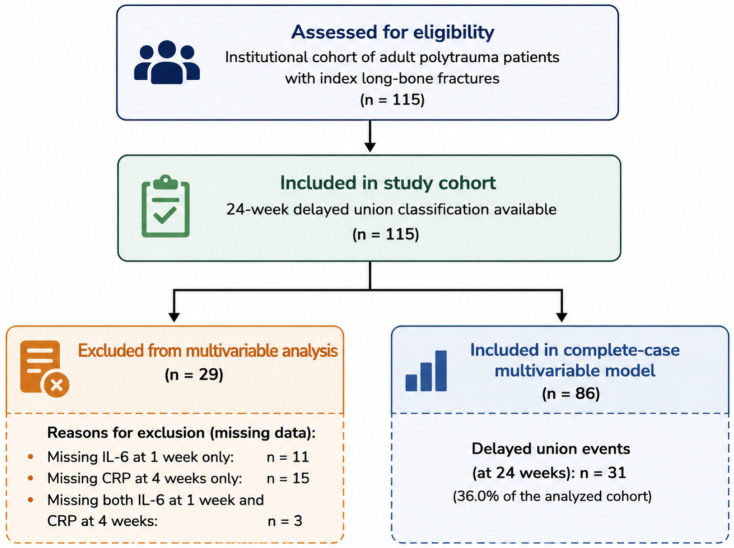
Flowchart of cohort derivation and complete-case multivariable-model inclusion.

Model-variable missingness affected IL-6 at 1 week in 14/115 patients (12.2%) and CRP at 4 weeks in 18/115 patients (15.7%); the overlap between these missing-data patterns was three patients. Accordingly, 29 patients were excluded from the complete-case multivariable model. Compared with complete-case included patients, excluded patients more frequently presented with shock on admission, whereas age, ISS, time to definitive fixation, delayed-union frequency, deep SSI, and reoperation rates were similar (Table 5). This pattern suggests that missingness may have been associated with physiologic severity rather than being fully random. Cohort derivation is shown in Figure 5. 

Sensitivity analyses excluding deep SSI cases, and excluding deep SSI and/or reoperation cases, are summarized in Table 6.

## 4. Discussion

The principal finding of this study is that persistent post-traumatic inflammatory activity in the subacute phase was associated with delayed bone healing after polytrauma. Among the evaluated biomarkers, CRP at 4 weeks showed the strongest and most reproducible association, whereas IL-6 demonstrated weaker unadjusted between-group differences that did not remain independently associated with delayed union in multivariable analysis. In contrast, fibrinogen did not discriminate between healing groups. Taken together, these findings support an association between persistent systemic inflammation and clinician-assigned delayed union, rather than a validated predictive role for serial inflammatory biomarkers.

From a clinical perspective, the present findings should not be interpreted as establishing predictive utility for CRP or IL-6. At most, persistently abnormal inflammatory markers may justify heightened clinical attention during follow-up in complex polytrauma cases. In the present dataset, an exploratory ROC-derived CRP threshold of 25.4 mg/L at 4 weeks showed moderate sensitivity and specificity, but this should not be interpreted as a clinically actionable cut-off without prospective validation. It should not be used in isolation to diagnose impaired healing and cannot replace serial clinical and radiographic assessment. Accordingly, CRP at 4 weeks should not be considered a sole marker for clinical decision making, but should be interpreted alongside the clinical presentation, serial imaging findings, and the presence or absence of postoperative complications such as infection and reoperation. Future prospective studies should evaluate whether biomarker trajectories add incremental value when combined with standardized radiographic scores and operative variables.

Our findings are biologically plausible in the context of current knowledge regarding post-traumatic immune activation and fracture repair [1,2]. Rather than the immediate inflammatory surge after injury, the more clinically relevant signal in this cohort appeared to be the persistence of inflammation into the subacute phase. In this respect, serial biomarker monitoring may provide a practical adjunct to radiographic follow-up [8].

Among the evaluated markers, CRP at 4 weeks showed the strongest observed association with delayed union. Importantly, CRP is a nonspecific inflammatory marker. In our dataset, CRP at 4 weeks was also higher in patients with deep SSI, reinforcing the concern that persistent CRP elevation may reflect postoperative infection or inflammatory burden rather than bone-healing biology alone. Nevertheless, sensitivity analyses excluding deep SSI cases, and excluding deep SSI and/or reoperation cases, did not abolish the association between CRP and delayed union. These analyses reduce, but do not eliminate, the risk of confounding. By contrast, IL-6 showed weaker and less robust findings: the between-group differences did not remain independent in the multivariable model, and exploratory longitudinal analyses did not demonstrate a significant delayed-union-by-time interaction. Earlier clinical observations in polytrauma patients with femoral fractures also suggested potential prognostic relevance of inflammatory biomarkers, including IL-6 [9]. Accordingly, the IL-6 results should be regarded as hypothesis-generating rather than confirmatory. Recent osteoimmunology reviews further support the concept that delayed union and nonunion reflect a dynamic interaction between immune signaling, angiogenesis, and local skeletal biology, and that prolonged inflammatory activation may shift fracture repair toward impaired healing rather than regeneration [10,11,12,13,14,15].

At the same time, the present results should be interpreted within the broader limitations of biomarker-based prediction. Recent reviews on fracture nonunion and biomarker research suggest that, although circulating markers are promising, no single laboratory parameter has yet achieved sufficient specificity to function as a standalone diagnostic tool for impaired healing [16,17,18,19,20]. This is particularly relevant in polytrauma patients, where biomarker trajectories may reflect not only osseous repair, but also the cumulative systemic burden of injury, surgery, transfusion exposure, and intercurrent complications. Our findings therefore support the use of CRP and IL-6 as adjunctive markers that may enrich clinical suspicion and guide surveillance, rather than replace radiographic and clinical assessment [17,18,19,20].

Another important issue is the operative context in which fracture healing occurs after major trauma. Modern polytrauma literature has moved from rigid damage-control versus early total-care dichotomies toward more individualized frameworks such as early appropriate care and safe definitive surgery [21,22,23,24,25]. These concepts emphasize that the timing and extent of fixation should be adapted to physiological status, associated injuries, and evolving systemic inflammation. In our cohort, a longer time to definitive fixation was associated with delayed union in univariable analysis, which is consistent with literature suggesting that fracture-timing decisions may influence downstream outcomes, even if the effect is strongly confounded by injury severity and overall patient condition [21,22,23,24,25,26]. Likewise, temporary external fixation and staged treatment should be interpreted within the logic of physiologic prioritization rather than as isolated mechanical variables.

From a practical standpoint, these considerations suggest that delayed healing after polytrauma is best understood through an integrated model rather than through any single predictor. Current literature on nonunion diagnosis, fracture-healing assessment, and emerging therapeutic strategies increasingly favors combining host factors, operative factors, imaging progression, and selected biologic markers into composite risk assessment frameworks [18,19,20,27,28,29,30]. Our study contributes to this direction by suggesting that persistently elevated CRP may merit further evaluation as one component of such frameworks, whereas the IL-6 signal in the present dataset remained weaker and hypothesis-generating. Future prospective studies should evaluate whether combining serial inflammatory markers with structured radiographic scores and more granular operative variables can improve early prediction of delayed union and support individualized surveillance strategies in polytrauma care [21,23,27,28,29,30].

The radiographic data reinforce the biological signal. Between-group differences in healing score were modest at 6 weeks, became clearer by 12 weeks in terms of bridged cortices, and were pronounced by 24 weeks. This is consistent with literature supporting RUST/mRUST-based systems as reproducible tools for following fracture healing over time [4,5,6,7].

The present study has several limitations. First, its retrospective design does not allow causal inference. Second, the cohort was moderate in size and included heterogeneous injury patterns and fixation strategies, which may have introduced residual confounding. Although fracture site, open-fracture grade, and AO/OTA classification were available descriptively, more granular biomechanical determinants of healing—such as fracture gap, reduction quality, fixation construct stability, and soft-tissue injury severity beyond open-fracture grading—were not captured in a standardized way. Accordingly, anatomical and mechanical heterogeneity may still have confounded the observed biomarker associations. In addition, although time to definitive fixation was incorporated into the analysis, the granularity of operative management variables remained limited, and differences in staged management, fixation construct, and mechanical environment may also have influenced healing outcomes. Third, the multivariable model was based on complete-case analysis and therefore included only 86 of 115 patients; excluded patients more frequently presented with shock on admission, indicating potential selection bias. Accordingly, complete-case analysis may have preferentially excluded more physiologically unstable patients, which could have biased the estimated association between CRP and delayed union and may limit generalizability to the sickest polytrauma patients. Fourth, deep surgical-site infection and reoperation were more frequent in the delayed-union group and may have influenced both inflammatory biomarker trajectories and fracture healing. Accordingly, CRP should not be interpreted as a bone-healing-specific marker, but rather as a clinically informative, nonspecific inflammatory signal. Because the source dataset did not retain the exact timing of deep surgical-site infection diagnosis or reoperation relative to the 4-week biomarker assessment, reverse causality cannot be excluded. Therefore, the sensitivity analyses excluding deep SSI cases and excluding deep SSI and/or reoperation cases should be interpreted as conservative robustness checks rather than as fully time-anchored causal analyses. Residual confounding likely remains, because tissue injury burden, fracture severity, fixation strategy, and the broader postoperative course may influence both CRP levels and delayed-union risk. Fifth, the events-per-variable ratio of 5.2 indicates that the multivariable model remains exploratory and potentially unstable despite acceptable apparent calibration and discrimination. Although collinearity was low, formal shrinkage estimation and penalized regression were not applied; therefore, some risk of overfitting may still remain. Sixth, the source dataset did not retain a prospective screening log for non-included patients, and blinding of radiographic assessors was not recorded. This may have introduced classification bias, because knowledge of the clinical context, postoperative course, or healing concerns could have influenced assignment of delayed-union status. In addition, because delayed union was analyzed as a clinician-assigned composite follow-up category without a prespecified radiographic threshold, the primary endpoint remains partly subjective and may be vulnerable to interobserver variability. This may reduce reproducibility and limit external validity. Despite these limitations, the study included serial biomarker measurements up to 4 weeks, sensitivity analyses addressing major postoperative confounders, and standardized radiographic follow-up up to 24 weeks, which support the clinical relevance of the observed associations.

The study has several strengths: a clinically relevant polytrauma cohort, serial biomarker sampling extending to 4 weeks, standardized radiographic follow-up out to 24 weeks with a secondary 9-month nonunion endpoint, and sensitivity analyses showing that the association between CRP and delayed union persisted after exclusion of patients with deep SSI and postoperative reoperation. These features support the clinical relevance of the observed association and justify prospective validation. Future studies with more granular operative data, predefined healing thresholds, and explicit adjustment for infection-related and reoperation-related events are needed to confirm the present findings.

## 5. Conclusions

In this exploratory retrospective cohort of polytrauma patients with long-bone fractures, persistent post-traumatic CRP elevation at 4 weeks was associated with clinician-assigned delayed union, whereas IL-6 findings were weaker and exploratory and fibrinogen showed limited discriminatory value. CRP should be interpreted as a nonspecific inflammatory signal that may reflect impaired healing, infection, reoperation, and/or postoperative inflammatory burden. These findings support association rather than validated prediction and require prospective validation with standardized outcome adjudication.

## Figures and Tables

**Table 1:** Baseline and early clinical characteristics according to delayed-union status. Abbreviations: BMI, body mass index; ISS, Injury Severity Score; SSI, surgical-site infection.

Variable	No Delayed Union (*n* = 76)	Delayed Union (*n* = 39)	*p*-Value
Age, years	46.3 ± 13.3	48.3 ± 11.2	0.363
Male sex	56/76 (73.7%)	30/39 (76.9%)	0.650
BMI, kg/m^2^	27.2 ± 4.2	28.3 ± 4.0	0.265
ISS	25.1 ± 4.8	26.1 ± 5.3	0.412
Shock on admission	18/76 (23.7%)	17/39 (43.6%)	0.047
Open fracture	15/76 (19.7%)	10/39 (25.6%)	0.626
Temporary external fixation	13/76 (17.1%)	12/39 (30.8%)	0.149
Time to definitive fixation, h	29.0 ± 14.0	35.3 ± 10.2	0.003
Deep SSI within 90 days	0/76 (0.0%)	4/39 (10.3%)	0.012
Reoperation by 6 months	4/76 (5.3%)	9/39 (23.1%)	0.010

**Table 4:** Logistic regression models for delayed union at 24 weeks. Abbreviations: OR, odds ratio; CI, confidence interval; CRP, C-reactive protein; IL-6, interleukin-6; EPV, events per variable. Model performance: apparent AUC = 0.80, optimism-corrected AUC = 0.75, Hosmer–Lemeshow *p* = 0.41.

Predictor	Univariable OR (95% CI)	*p*-Value	Adjusted OR (95% CI)	*p*-Value
Age, years	1.01 (0.98–1.04)	0.414	1.02 (0.98–1.06)	0.454
Shock on admission	2.49 (1.09–5.68)	0.030	0.95 (0.27–3.37)	0.934
Open fracture	1.40 (0.56–3.50)	0.469	0.80 (0.22–2.98)	0.744
Time to definitive fixation (per 12 h)	1.58 (1.08–2.29)	0.017	1.54 (0.89–2.68)	0.122
IL-6 at 1 week (per 10 pg/mL)	1.13 (0.98–1.29)	0.091	1.11 (0.92–1.34)	0.286
CRP at 4 weeks (per 10 mg/L)	2.40 (1.58–3.64)	<0.001	2.16 (1.36–3.43)	0.001

**Table 5:** Comparison of patients included in and excluded from the complete-case multivariable model.

Variable	Included (*n* = 86)	Excluded (*n* = 29)	*p*-Value
Age, years	46.5 [40.2–58.0]	43.0 [39.0–50.0]	0.110
ISS	25.0 [21.0–27.8]	28.0 [22.0–31.0]	0.055
Shock on admission	21/86 (24.4%)	14/29 (48.3%)	0.029
Open fracture	18/86 (20.9%)	7/29 (24.1%)	0.919
Time to definitive fixation, h	30.2 [22.6–40.2]	30.5 [24.5–40.0]	0.638
Delayed union at 24 weeks	31/86 (36.0%)	8/29 (27.6%)	0.545
Deep SSI within 90 days	3/86 (3.5%)	1/29 (3.4%)	1.000
Reoperation by 6 months	10/86 (11.6%)	3/29 (10.3%)	1.000

Abbreviations: ISS, Injury Severity Score; SSI, surgical-site infection. Continuous variables are shown as median [interquartile range]. *p*-values were calculated using the Mann–Whitney U test or Fisher’s exact test, as appropriate.

**Table 6:** Sensitivity analyses for the association between CRP at 4 weeks and delayed union.

Analysis	*n*	Delayed-Union Events	Adjusted OR for CRP at 4 Weeks (per 10 mg/L)	*p*-Value
Primary complete-case model	86	31	2.16 (1.36–3.43)	0.001
Excluding deep SSI	83	28	2.02 (1.26–3.26)	0.004
Excluding deep SSI and/or reoperation	75	23	1.92 (1.15–3.19)	0.012

All models were adjusted for age, shock on admission, open fracture, time to definitive fixation, and IL-6 at 1 week. Abbreviations: CRP, C-reactive protein; OR, odds ratio; SSI, surgical-site infection.

## Data Availability

The data presented in this study are available from the corresponding author upon reasonable request, subject to institutional and ethical restrictions.
